# Management of severe hypospadias

**DOI:** 10.4103/0970-1591.40621

**Published:** 2008

**Authors:** Massimo Catti, Delphine Demède, Anne-Frédérique Valmalle, Pierre-Yves Mure, Frédéric Hameury, Pierre Mouriquand

**Affiliations:** Department of Pediatric Urology, Debrousse Hospital, Claude-Bernard University, Lyon, France

**Keywords:** Hypospadias, Urethral plate, Chordee, Fistula, Dehiscence, Stenosis, Urethrocele, Duplay urethroplasty, Mathieu urethroplasty, Onlay urethroplasty, Koyanagi urethroplasty, Buccal graft urethroplasty, Bracka urethroplasty

## Abstract

Many classifications of hypospadias have been published, mainly based on the position of the ectopic meatus, which is an insufficient criterion to define the severity of this malformation. What really marks the proximal landmark of this malformation is the level of division of the corpus spongiosum, which is always proximal to the ectopic meatus. In this article, we will focus on the most severe forms of hypospadias which include those with a proximal division of corpus spongiosum (below the midshaft), important chordee and a poor development of the ventral radius, reflecting a marked hypovirilization of the genital tubercle, and cripple hypospadias resulting from several previous failed surgical procedures. The principle of hypospadias surgery will be reviewed together with the outcome of the current surgical techniques. Furthermore, common complications will be outlined. There is no minor or major hypospadias and all forms require a solid experience of the surgeon, as minor looking hypospadias may turn out to be far more complex to repair than they appear once the ventral radius of the penis has been dissected.

## DEFINITION AND ANATOMY

A possible definition of hypospadias is the incomplete virilization of the genital tubercle causing an insufficient development of the tissues forming the ventral aspect of the penis.

Three associated anomalies are classically found in hypospadias: an ectopic position of the urethral meatus, a ventral curvature of the penis (chordee), and a defect of the ventral prepuce. A hypospadiac penis presents, from the tip to the base: a ventrally opened glans penis, an absent frenular artery and a missing segment of urethral tube which is replaced by a urethral plate extending from the ectopic meatus up to the glans cap. The tubular urethra proximal to the ectopic meatus is hypoplastic, not surrounded by any corpus spongiosum and covered by a thin layer of skin tightly stuck on it.

The division of the corpus spongiosum is always proximal to the ectopic meatus and is often outlined on the ventral skin by a small cutaneous ridge. Proximally to the division of the corpus spongiosum, all structures forming the ventral aspect of the penis are normal as well as the dorsum.

**Figure 1:**
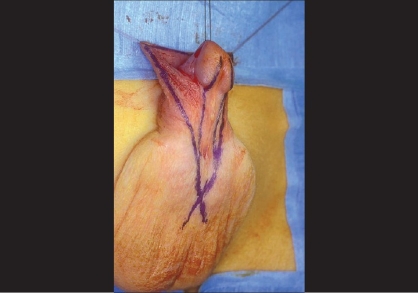
Division of corpus spongiosum

Consequently, what really marks the proximal landmark of this malformation is not the position of the ectopic meatus, but the level of division of the fan-shaped corpus spongiosum which represents the summit of a ventral triangular defect, the two sides being represented by the two lateral pillars of atretic spongiosum and the basis by the glans plate. This triangle is easily outlined by drawing two lines following on each side the junction between the inner and outer aspects of the prepuce. Where the two lines cross is where the corpus spongiosum splits [Figure 1].

The penile ventral curvature is the direct consequence of the hypoplastic tissues laying in the ventral triangular defect described above. The thin ventral skin is often very adherent to the underlying hypoplastic urethra. Its freeing (penile degloving) sorts out the curvature in most cases. The hypoplastic pillars of spongiosum extending upward in a fan-shaped situation on each side of the urethral plate can create a true chordee (i.e., bands of fibrous tissue). Rarely, the ventral curvature of the penis is due to a true asymmetrical development of the corpora cavernosa.

Many classifications of hypospadias have been published, mainly based on the position of the ectopic meatus, which is an insufficient criterion to define the severity of this malformation.

Three main types of hypospadias may be practically distinguished: those with a distal division of the corpus spongiosum with little or no chordee; those with a proximal division of the corpus spongiosum with chordee and a poor development of the ventral aspect of the penis (ventral radius); and, finally, those who underwent several previous failed surgical procedures (hypospadias cripple) which present with scarred tissues, inadequate urethra, abnormal meatus, fistula, and dehiscence of various severity.

**Figure 2:**
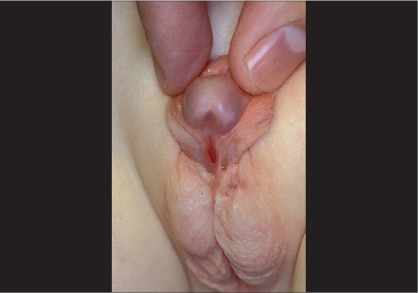
Severe hypospaduas

In this article, we will focus on the most severe forms of hypospadias which reflect a marked hypovirilization of the genital tubercle [Figure 2].

There is no minor or major hypospadias, as all forms of hypospadias require a solid experience of this surgery. Some minor looking hypospadias may turn out to be far more complex to repair once the ventral radius of the penis has been dissected.

Severe hypospadias might be somehow more straightforward to repair as from the beginning of the operation the surgeon knows what to expect.

## SURGICAL PRINCIPLES

The three steps of this reconstruction are to straighten the penis by correcting the chordee, to refashion the missing urethra (urethroplasty) placing the urethral meatus at the tip of the glans and giving it a slit-shaped opening, and to rebuild the ventral aspect of the penis to get a normal looking penis with or without circumcision. This reconstruction should allow the child to have micturitions standing up without pushing with a straight and wide stream and to access in adulthood to a satisfactory sexual life with straight erections.

### Straightening the penis

Fully degloving the penile skin shaft, in most cases, is a sufficient maneuver to straighten the penis. The freeing of all ventral tissues is usually performed before deciding if complementary procedures are necessary to straighten the penis. If the penile curvature persists, freeing the urethral plate from the corpora cavernosa down to the normal urethra is an alternative advocated by some. In <5% of cases, chordee still persists after these maneuvers and a dorsal plication of the corpora cavernosa following Nesbitt principle is then needed. Some authors argue that separating the urethral plate from the underlying corpora cavernosa may jeopardize its blood supply and prefer to perform a Nesbitt procedure or one of its' variants straightaway.

Experienced surgeons perform erection tests only when a doubt persists after this dissection. Extensive dissection of the ventral tissues usually allows achieving penile rectitude even in the most severe forms of hypospadias as demonstrated by the Koyanagi procedure where dorsal plication of the corpora is rarely needed.

### Urethroplasty

Once the chordee is corrected and possibly checked by an artificial erection test, the urethroplasty is then performed.

The choice of the technique depends on the quality and width of the urethral plate.

If it is healthy and wide, it can be tubularized following the Thiersch-Duplay procedure.[1]

If it is healthy but narrow, various options exist. One of the most popular in these days is the Snodgrass procedure or tubularized incised plate urethroplasty (TIP)[2] where the urethral plate is incised longitudinally in order to make it wide enough to be subsequently tubularized following the Thiersch-Duplay procedure.

**Figure 3:**
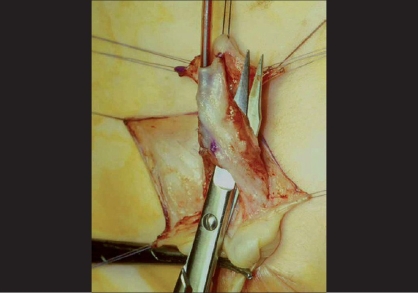
Onlay flap

**Figure 4:**
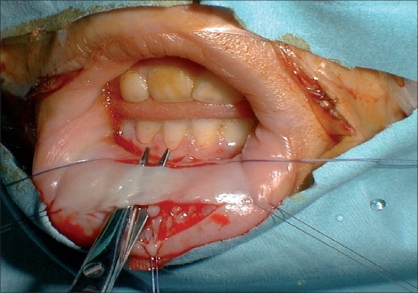
Harvesting buccal mucosa from lower lip

Alternatively, a rectangle of tissue can be sutured to the lateral edges of the urethral plate in order to bring additional tissue to replace the defective ventral part of the urethra (Onlay urethroplasty,[3] [Figure 3]). This rectangular flap of tissue may be either harvested from the inner aspect of the prepuce with its pedicle (Onlay island flap procedure[3]) or is a free graft of buccal mucosa[4] [Figure 4].

Other free grafts, such as bladder mucosa[5] or skin,[6] are nowadays abandoned by the most. Another option is to complete the Snodgrass procedure by grafting the raw dorsal area of the urethral plate with buccal mucosa (In-lay or Snod-graft procedure, as described by Barbagli *et al*.[7]) to create a wide urethral plate subsequently duplay-ed.

**Figure 5:**
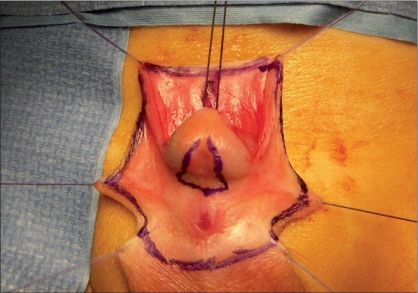
Incision marking for Koyanagi

When the urethral plate is not preservable, a full substitution of the missing urethra must be performed, either by creating a tube with buccal mucosa or with a rectangle of inner prepuce which is pediculized down to the base of the penis and transferred to its ventral aspect (Asopa-Duckett procedure[8]); or by using the Koyanagi procedure[9] [Figure 5], where the urethral plate, the adjacent tissues and the inner aspect of the preputial hood are extensively mobilized with their blood supply and transferred to the ventral aspect of the penis to reconstitute a wide and healthy urethral plate which is subsequently duplay-ed.

### Reconstruction of the ventral radius of the penis

Once the penis is straight and the urethra fully reconstructed, many advocate covering the neourethra with some healthy surrounding tissues coming either from the corpus spongiosum lying laterally (spongioplasty) or from subcutaneous scrotal, or dorsal penile tissues. Further steps include the reconstruction of the neo meatus (meatoplasty), the ventral aspect of the glans (glanuloplasty), and the creation of a mucosal collar around the glans.

Finally, the penile skin cover may be performed with or without preservation of the foreskin. In most cases of severe hypospadias, a circumcised penis will be obtained.

## CURRENT TECHNIQUES IN SEVERE HYPOSPADIAS

### Proximal hypospadias

Extra tissue is often needed to reconstruct the missing urethra in these cases.

The first choice for many is a pediculized flap which is harvested from the inner aspect of the dorsal preputial hood and transferred ventrally (Onlay procedure). The urethral plate is used as a mooring plate and constitutes the roof of the neourethra, the rectangle of pediculized preputial mucosa is then stitched along the edges of the urethral plate using fine resorbable sutures (6-0 or 7-0 polydioxanone or polyglactin). The Onlay technique avoids circular anastomoses and therefore prevents strictures at the level of the proximal anastomosis. Baskin *et al*.[10] reported 5-10% of fistulas, 1% of meatal stenosis, and 0.5% of urethrocele, while Elbakry[11] reported complications in 42% of the patients, including breakdowns in 7%, fistulas in 23%, urethral strictures in 9%, and diverticula in 4%.

The classical Asopa-Duckett procedure ignores the urethral plate, which is excised. A pediculized preputial tube of foreskin is placed between the ectopic meatus and the glans. Because of the circular anastomosis, the risk of stricture is higher compared to Onlay procedure. The complication rate varies a lot in the literature, but is certainly one of the highest. In our series of 84 patients[12] with Onlay procedure, 15% had fistulas and 6% of them requiring a secondary procedure. The fistula rate reached 20% in redo procedures, while no stenosis was noted. Mollard and Castagnola[13] in their series of 92 cases reported 19 complications in 15 patients (14 fistulas, 3 meatal stenosis, and 2 urethroceles). Ransley *et al*.[14] reported fistulas in 34%, urethral strictures in 12%, meatal stenoses in 18%, and a redo urethroplasty was performed in 50% of the patients.

The Snodgrass procedure has also been also applied for proximal hypospadias in the absence of severe penile curvature (which sounds somewhat contradictory) and when the urethral plate is supple and healthy. In their own series of 33 posterior hypospadias, Snodgrass and Lorenzo[15] reported 33% of complications, of whom 21% being fistulas. Samuel and Wilcox[16] reported 1 fistula in 18 patients and also claimed better cosmetic results.

Alternatively, a “snodgraft” or inlay procedure may be used to secure the quality of the urethral plate as described above. Barbagli *et al*.[17] reported a success rate of 85% in their series.

With the Koyanagi repair and its modifications a long and wide strip is harvested from the urethral plate and adjacent tissues and the preputial hood, and subsequently duplayed. Koyanagi *et al*.[18] reported a complication rate of 47% with good cosmetic results. In our experience, the Koyanagi technique is at its best in the most proximal hypospadias. We noticed in our first 20 cases, a significant difference of compliance between the native and the reconstructed urethra sometimes leading to the development of a proximal urethrocele and dysuria.

An alternative is the use of free grafts to reconstitute the defective urethral plate. Skin had a poor track record as a urethral substitute, especially extra-genital skin. The use of bladder mucosa graft had a period of popularity both in primary and redo procedures. However, long-term results were disappointing with many complications such as meatal stenosis and urethral prolapse, requiring additional procedures in 66% of the cases.[19] A free graft of buccal mucosa taken from the inner aspect of the cheek or the inferior lip is actually the most commonly accepted option. As long as the harvesting site is far from the lip bordures, lip commissures and Stenon's duct, little morbidity is observed with this technique.

In a 5-year follow-up of 22 buccal mucosal urethroplasties, Dessanti *et al*.[20] reported 32% of complications, mainly strictures, with 13% of the patients being reoperated. Duckett *et al*.[21] reported complications in 57% of his patients, including five meatal stenosis, seven strictures, two fistulae, and one breakdown. Results are better when the graft is used as a patch rather than as a tube, with a lower risk of stricture.

## CRIPPLE HYPOSPADIAS

There are no standardized techniques for redo procedures as the experience of the surgeon is of paramount importance to adjust the most appropriate technique to each case. A preoperative course of injectable βHCG or testosterone, or local dihydro testosterone cream may be helpful to improve tissular vascularization.

It is actually questionable whether androgen treatment helps healing.

Chordee must to be checked again and if persistent needs to be corrected following the same steps as mentioned above. Buccal mucosa is commonly used for redo urethroplasties when a circumcision was previously performed or when the ventral tissues are too scarred to be reused. In distal breakdowns, salvage Mathieu or Koff procedures are potential options.

The TIP urethroplasty has been reported[22] to have a successful outcome in selected cases with a persistently supple urethral plate after previous failed procedures. However, it should be avoided if the urethral plate is obviously scarred.

Composite urethroplasties might be needed in complex cases.

Complication rate (mainly fistulas and urethral dehiscence) for buccal graft redo urethroplasties is of 30% in our experience[23] and of 20% according to Baskin and Duckett.[24] However, secondary Duplay procedure is often possible after a dehisced buccal graft urethroplasty.

## MULTISTAGE PROCEDURES

Multistage procedures find their best indications in multioperated hypospadias and, according to some, in proximal hypospadias when the urethral plate cannot be preserved.

Stage-one repair includes an extensive dissection of the ventral aspect of the penis from the ectopic meatus up to the glans which is widely opened. A rectangle of inner prepuce or buccal mucosa is grafted onto the ventral aspect of the penis with multiple fine stitches and subsequently immobilized with a “tie-over” pressure dressing. Six months after, the stage-two repair consists of the tubularization of the previously created urethral plate.

**Figure 6:**
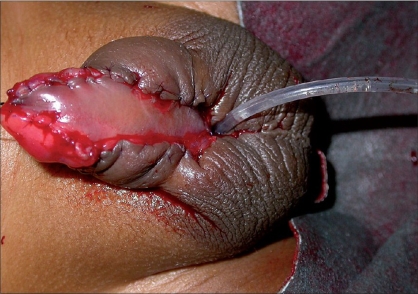
Buccal mucosal graft

Bracka[25] reported better cosmetic results using two-stage procedures [Figure 6], inspired by Cloutier's technique.[26] In his personal series,[25] he reported fistulas in 5.7% and strictures in 7% of the cases.

Using skin to replace the urethra raises some concerns as it exposes to a higher risk of stricture and provides poorly compliant neourethra. Buccal mucosa seems to provide better long-term results as shown by Barbagli *et al*.[27]; in his series, buccal mucosa grafts provided 81% of success in one-stage procedures and 82.3% in multistage procedures, while penile skin grafts 80% and 50%, respectively.

## POSTOPERATIVE MANAGEMENT

The use of a urethral catheter in order to drain urine and to caliber the newly reconstructed urethra is advocated by most, although no consensus exists about necessity and duration of urethral stenting. Equally, the use of antibiotics is controversial. Wound-dressing and postoperative cares are of paramount importance in order to minimize the risks of infections and wound dehiscence. It appears essential to keep the penis still during the first healing phase to improve the outcome and reduce the child's discomfort.

**Figure 7:**
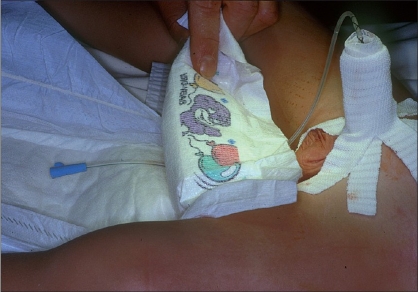
Dressing

In our center, we use for these cases a soft silicone stent for at least 10 days, with intravenous intraoperative antibiotics followed by oral prophylaxis until the removal of the catheter. A “daisy dressing” [Figure 7] is applied on the penis, changed on the fourth postoperative day and definitely removed with the stent. This dressing includes the application of sulfadiazine and hyaluronate cream on the wound, a Mepitel® mesh and an elastic gauze maintained by Elastoplast®.

## COMPLICATIONS

Complications are common and should be treated at least 6 months after the initial procedure in order to let tissues recover properly.

### Unsatisfactory cosmetic results

The surgeon's and the patient's points of views on the cosmetic result are often very different.[28] Unsatisfactory cosmetic results are quite common with irregular suture lines, skin blobs, and redundant skin forming a jabot. Disappointing results are often seen when the ventral aspect of the glans is too short and when there is no mucosal collar around the glans.

### Fistulas

Fistulas are the second most common complication leading to an abnormal stream or drops coming from the reconstructed urethra, proximal to the neo-meatus.

The incidence of fistulae varies with the technique and is more frequent with free graft than pediculized grafts.[12] For most one-stage repairs, the reported rate is between 10 and 15%.[29] In our experience, the Onlay procedure has a 15% fistula rate going up to 20% in cripple hypospadias.[30]

Although late fistulae exist, they are usually early complications that may spontaneously heal when small and not sustained by a urethral stricture. If a small fistula persists 6 months after the initial procedure, it should be excised, sutured, and covered with several layers of healthy surrounding tissues, after ruling out a distal urethral stricture.

Larger fistulas are rarer and usually reflect an inadequate reconstruction with infectious or vascular complications. In these cases, a redo urethroplasty is necessary.

Fistulas may have variable locations, most commonly being proximal to the glans corona in a lateral position. These are not easy to close and tend to recur if a simple excision-coverage procedure is performed. A complete redo urethroplasty is often preferable and the Mathieu urethroplasty may be helpful.

### Strictures

Urethral strictures are rarer nowadays as circular anastomoses are less common. Meatal stenoses are treated with a meatoplasty, as repeated dilatations are badly accepted by children. Proximal stenoses require a redo urethroplasty.

Ongoing urethral urine flow impairment may lead to an abnormal bladder behavior with high-pressure voiding and possibly upper urinary tract damage. It is, therefore, important to recognize and treat urethral strictures promptly. Urine flow studies are not reliable to assess the urethral caliber as they often prove to be abnormal even if the reconstruction is satisfactory.

### Mucosal ectropion

Mucosal ectropion was commonly observed with bladder mucosal graft urethroplasties and often associated with pseudopolyps requiring a resection. Recurrence is common as well as secondary meatal stenosis. These reasons led to the abandon of bladder mucosal grafts.

### Balanitis Xerotica Obliterans

Balanitis xerotica obliterans is a rare complication related to a chronic inflammation and fibrosis of the meatus and glans. Meatoplasty or redo urethroplasty using buccal mucosa may be required when topical steroids fail.

### Urethrocele

Urethrocele is often related to the difference of tissular compliance between the native and the reconstructed urethra. In our experience, the Koyanagi procedure is often associated with this complication. Supporting the reconstructed urethra with several layers of well-vascularised tissues may minimize the difference of tissular elasticity. The solution is often a redo urethroplasty with excision of the redundant urethral tissues after excluding a distal stricture.

### Hairy urethra

Hairy urethra should no longer be seen with modern procedures although it may complicate the Koyanagi procedure, where peno-scrotal skin is used to reconstruct the proximal urethra. Urethral stones may develop in the hairy segment of the urethra. A redo urethroplasty excising the hairy portion is then required.

### Meatal regression or glanular dehiscence

These complications, quite frequent after a MAGPI or a Koff procedure, should be prevented by the adequate mobilization of the glans wings before their suture together on the midline. A complementary urethroplasty is often required.

### Persistent chordee

Persistent chordee is quite rare and poorly reported.

Literature data about the long-term outcomes of corporal dorsal plication is lacking. It might be that some dorsal corporeal plication might lead to subsequent penile deformities in adulthood.

### Disasters-cripple hypospadias

Cripple hypospadias are the results of multiple failed interventions, sometimes leading to complete disasters with persistent severe chordee, fibrous and scarred tissues, irregular skin blobs, and large fistulas partially covered by skin bridges.

Cripple hypospadias is often the result of traumatizing dissection, poor tissular vascularization, sutures under tension, inappropriate urine drainage, infections, or misdiagnosis (intersex).

## TECHNICAL TIPS

Most surgeons use magnification glasses for this surgery. Endless debates exist about the use of antibiotics, tourniquets, types of urine drainage, dressings, and sutures. Each surgeon has his or her own habits and each needs to compare honestly his or her results with others.

Surgical repair should be carried out starting from 6 months of age and if possible, before 2 years, as children under 2 years of age have reflex micturitions which facilitate voiding after surgery.

The current trend is to keep these children in the hospital for as short a time as possible to reduce the psychological impact of being in hospital. Here again, it seems that the best age for surgery is probably between 12 and 24 months. Data are again lacking and opinions may be very variable.

Preoperative penile hormonal stimulation is debated as there is no agreement about the dose and type of stimulation to be used. Systemic βHCG and testosterone, local dihydrotestosterone or testosterone cream, epidermal growth factor, and growth hormone are regularly discussed as means to improve the size of the penis and its ability to heal. Little is known about the long-term effects of these hormonal treatments, particularly concerning growth and precocious puberty.

## LONG-TERM OUTCOMES

Results of hypospadias surgery can be analyzed using both subjective and objective criteria.

Objective criteria include functional evaluation of micturition. Uroflowmetry is difficult to interpret as its profile is often abnormal even if reconstruction is satisfactory. These flat profiles often reflect the poor compliance of the reconstructed urethra or the abnormal bladder behavior after hypospadias reconstruction.

Subjective criteria are more difficult to define and evaluate, but certainly include cosmetic appearance, psychosocial adjustment, sexual function, and body image.

A few publications are reported with modern procedures as long-term results available to date mostly concern procedures that have been abandoned. Overall, more than 30% of posterior hypospadias patients were reported to have complex long-term problems.[31]

Khoury[32] showed that 69% of TIP urethroplasties had normal urinary peak flow rates and 46/48 patients had a postvoiding residue inferior to 10%. Snyder *et al*.[33] found normal uroflow rates in patients undergoing Onlay or Duckett procedures with a follow-up of 14 years, which does not match with other published data. In a review of 44 patients undergoing two-stage repairs, 40% complained of urinary spraying and 40% milked their urethra after voiding.[34] Similar results were noted after buccal mucosa grafts, with 26% describing problems such as urinary spraying and stream deviation.[35]

An objective assessment of the cosmetic appearance is difficult. In a recent study, the TIP urethroplasty was felt to have the most cosmetically appealing when compared with Mathieu and Onlay procedures.[36] Lam *et al*.[34] reported that 92% of patients undergoing a two-stage repair were satisfied and 88% considered their penis to be normal. In contrast, Nelson,[35] reviewing single-staged buccal urethroplasties reported only 28% of satisfaction with the appearance of the penis.

The long-term psychological and sexual implications were often neglected in the past. Very few long-term studies have been published,[28,37,38] mainly showing that the sexual life of these patients is often normal, although starting later than controls. However, when compared to controls, hypospadias patients were found to be more inhibited in seeking sexual contacts, were significantly less sexually active with fewer sexual partners and were significantly less satisfied with their sexual life.[38]

Fertility is not affected unless the hypospadias is associated with undescended testes.

## CONCLUSIONS

Hypospadias surgery remains very challenging, with a significant rate of complications even in the best hands. This surgery should be performed by experienced pediatric urologists performing a minimum of 40-50 urethroplasties per year.

Pre- and perioperative treatments adjusting the healing capacity of the patient are promising fields of research which may improve the outcome. The main challenge is to find an adequate tissue to substitute the missing urethra, as neither skin nor buccal mucosa nor bladder mucosa are entirely satisfactory. Tissue engineering[39] may be a promising avenue to provide urethral material for urethroplasty, although practical evidence is still awaited.
